# Serotonin Receptors Expressed in *Drosophila* Mushroom Bodies Differentially Modulate Larval Locomotion

**DOI:** 10.1371/journal.pone.0089641

**Published:** 2014-02-25

**Authors:** Bryon Silva, Nicolás I. Goles, Rodrigo Varas, Jorge M. Campusano

**Affiliations:** 1 Departamento de Biología Celular y Molecular, Facultad de Ciencias Biológicas, Pontificia Universidad Católica de Chile, Millennium Nucleus Stress and Addiction, Santiago, Chile; 2 Facultad de Ciencias de la Salud, Universidad Autónoma de Chile, Talca, Chile; Vlaams Instituut voor Biotechnologie and Katholieke Universiteit Leuven, Belgium

## Abstract

*Drosophila melanogaster* has been successfully used as a simple model to study the cellular and molecular mechanisms underlying behaviors, including the generation of motor programs. Thus, it has been shown that, as in vertebrates, CNS biogenic amines (BA) including serotonin (5HT) participate in motor control in *Drosophila*. Several evidence show that BA systems innervate an important association area in the insect brain previously associated to the planning and/or execution of motor programs, the Mushroom Bodies (MB). The main objective of this work is to evaluate the contribution of 5HT and its receptors expressed in MB to motor behavior in fly larva. Locomotion was evaluated using an automated tracking system, in *Drosophila* larvae (3^rd^-instar) exposed to drugs that affect the serotonergic neuronal transmission: alpha-methyl-L-dopa, MDMA and fluoxetine. In addition, animals expressing mutations in the 5HT biosynthetic enzymes or in any of the previously identified receptors for this amine (5HT_1A_R, 5HT_1B_R, 5HT_2_R and 5HT_7_R) were evaluated in their locomotion. Finally, RNAi directed to the *Drosophila* 5HT receptor transcripts were expressed in MB and the effect of this manipulation on motor behavior was assessed. Data obtained in the mutants and in animals exposed to the serotonergic drugs, suggest that 5HT systems are important regulators of motor programs in fly larvae. Studies carried out in animals pan-neuronally expressing the RNAi for each of the serotonergic receptors, support this idea and further suggest that CNS 5HT pathways play a role in motor control. Moreover, animals expressing an RNAi for 5HT_1B_R, 5HT_2_R and 5HT_7_R in MB show increased motor behavior, while no effect is observed when the RNAi for 5HT_1A_R is expressed in this region. Thus, our data suggest that CNS 5HT systems are involved in motor control, and that 5HT receptors expressed in MB differentially modulate motor programs in fly larvae.

## Introduction

Biogenic amines (BAs) play an important role in the generation or modulation of several behaviors, including locomotion. For instance, it has been shown that dopamine (DA) containing neurons that constitute the nigro-striatal dopaminergic pathway are key players in the planning and execution of motor programs in vertebrates. The importance of DA-containing neurons is evident in Parkinson’s disease, where the progressive death of the dopaminergic neurons that form the nigro-stratal pathway is the cellular event responsible for the expression of the clinical signs of the disease [1].

BA systems are highly conserved throughout evolution and several data obtained in different animal models, in particular the fly *Drosophila melanogaster*, suggest that neuronal pathways containing BAs in invertebrates would play similar roles to those described in vertebrates. For example, it has been shown that adult flies expressing a mutation in the tyrosine hydroxylase (TH) gene, the rate-limiting enzyme in DA biosynthesis, show important locomotor impairment [2]. In addition, activation of fly DA neurons by optogenetic tools increases locomotion, consistent with the idea that this amine contributes to motor activity [3]. On the other hand, little or no locomotor activity is observed in adult animals expressing a mutation for a biosynthetic enzyme common to two other neuroactive amines, octopamine (Oct) and its precursor tyramine (Tyr), which suggest these BAs also regulate locomotion [4]. Moreover, injection of female flies with a different BA, serotonin (5HT), induces a dose-dependent increase in locomotor activity [5]. These behavioral data are consistent with genetic information obtained by Jordan et al, 2006 [6] who carried out a quantitative trait loci analysis to identify genes associated to locomotor behavior in adult flies, including *tyr1*, a gene affecting catecholamine biosynthesis, *Catsup*, a negative regulator of TH activity, and Dopa decarboxylase (*Ddc*), an enzyme associated to DA and serotonin (5HT) biosynthesis [6]. Thus, the available data support the idea that several BAs would play a role in the execution or modulation of motor programs in flies.

The contribution of different BAs to locomotion is also evident in flies at the larval stage. It has been shown that larvae expressing mutations in the gene coding for the vesicular monoamine transporter (*DvMAT*), the protein responsible for vesicle storage of BAs, show reduced locomotion [7], which is consistent with data obtained in adult flies that showed increased locomotion after overexpression of this protein in aminergic neurons [8]. Some reports have also shown that locomotion is greatly decreased in larvae expressing mutations in the genes responsible for Oct and Tyr biosynthesis [9], [10], consistent with data obtained in adult flies expressing mutations for these biosynthetic enzymes [4]. Also consistent with the proposition that BAs play a role in the generation of locomotor behavior in larvae, Rodriguez Moncalvo and Campos, 2009 [11] recently showed that the larval motor response associated to a light stimulus is affected when the Ddc-expressing neurons are inactivated, an effect that depends on 5HT containing neurons localized in larval brain hemispheres. Therefore, several evidences suggest the importance of BA systems in the generation of motor behavior in flies both at the larval and adult stages.

Different studies carried out mostly in adult flies suggest that two brain regions are responsible for the generation and/or modulation of motor programs in *Drosophila*: the Central Complex (CC) and the Mushroom Bodies (MB). Thus, it has been postulated that while the CC is the region of the adult fly brain involved in the generation of motor programs [12], [13], the MB would play a modulatory role, inhibiting these programs [14]–[16]. *Drosophila* larvae also develop motor programs that are possible to be studied in detail [17]–[19]. Although the lower complexity of the larval brain makes it an ideal system to dissect out the contribution of specific neural systems to these programs, the amount of information available on this issue is limited. Nevertheless, it has been shown that, as in the adult animal, the CC is important in the generation of motor programs in fly larvae [20]. Moreover, a recent report suggests that the MB would be the sole responsible for the aversive response of fly larvae to a light stimulus [21]. Overall, these data suggest that both CC and MB participate in the generation of motor programs in fly larvae, as it has been shown in adult animals.

Interestingly, these two insect brain regions receive extensive innervation from neurons containing BAs [22], [23]. Thus, although the neural substrate for the effects of BAs on fly locomotion is far from being fully understood, it is possible to suggest that aminergic systems participate in the generation or modulation of motor programs by modifying the activity of CC and MB intrinsic neurons. This is a proposition that has not been extensively evaluated.

In our lab we are interested in assessing the contribution of specific brain regions to the generation of motor programs in flies, and how those programs are modulated by BA systems. Here we evaluate whether 5HT and its receptors expressed in *Drosophila* MB differentially affect larval locomotion. Four receptors for 5-HT have been described in *Drosophila*: one shares sequence homology to the vertebrate 5HT receptor type 2 (5HT_2_R; CG1056), one is similar to the vertebrate serotonin receptor type 7 (5HT_7_R; CG12073) and two show high homology to vertebrate 5HT_1_ type receptor (5HT_1A_R and 5HT_1B_R; CG16720 and CG15113, respectively). Our results show that the different 5HT receptors differentially modulate locomotion in *Drosophila* larvae.

## Materials and Methods

### Fly Stocks and Crosses

Flies were maintained in vials containing a standard agar medium at 19°C under a 12/12 h light/dark cycle.

The mutant flies used in this work were obtained from the Bloomington *Drosophila* Stock Center (BDSC, Indiana University, IN, USA), except for the *Ddc* ^ts2^/CyO mutant fly [24] which was originally part of the O’Dowd Lab fly stock (University of California Irvine, CA, USA). All flies were cantonized. The list of mutant flies is as follows: *w* ^1118^;Trh^c01440^ (BDSC line #10531; [25]); *y* ^1^ *v* ^1^;5HT_1A_R^EY09988^ (line #17629; [26]); *w* ^1118^;5HT_1B_R^MB05181^ (line # 24240; [26]); *y* ^1^ w*;5HT_2_R^MI00459^/TM6B,Tb^1^ (line # 31012; [26]); *w* ^1118^;5HT_7_R^f05214^ (line # 18848; [25]); and y^1^w^67c23^;mbm^EY19304^ (line # 23103; [26]). Wildtype strains used were Canton-S, *y* ^1^ *v* ^1^ and *w^1118^*.

For specific experiments, the following Gal4 drivers were used: the pan neuronal elav-Gal4 (on X; [27]); the general MB driver w*;OK107-Gal4 [28]; the MB γ-lobe specific *w* ^1118^;201y-Gal4 [29], all originally part of the O’Dowd Lab stock; and the MB α’/β’-lobe specific c305a-Gal4 (generous gift of Dr Hiromu Tanimoto, Max Planck Institute of Neurobiology, Martinsried, Germany; [30]).

The UAS-RNAi lines directed to the different 5HT receptors were: y^1^v^1^;UAS-RNAi^5HT1AR^ (BDSC; line # 25834); y^1^v^1^;UAS-RNAi^5HT1BR^ (BDSC; line # 25833); y^1^v^1^;UAS-RNAi^5HT2R^ (BDSC; line # 31882); and y^1^v^1^;UAS-RNAi^5HT7R^ (BDSC; line # 27273) [31].

Male flies containing a specific UAS-RNAi element were crossed overnight to virgin females flies containing a Gal4 driver. Vials containing new animals from these crosses were kept at 19°C, to diminish the effects of Gal4-driven genes on development [32]. Animals were brought to room temperature (24–25°C) at least one day before the beginning of an experiment. Animals containing one copy of the Gal4 or UAS transgenes were cantonized and used as genetic controls. In the case of the experiments with the *Ddc* ^ts2^/CyO mutant fly, the animals were incubated for 20 min at 32°C and then tested at room temperature.

### Assessment of RNAi Efficiency

Semi-quantitative RT-PCR procedure (previously described in Campusano et al, 2005 [33]) was used to evaluate the efficiency of the RNAi for the different transcripts. Briefly, fly males containing the UAS-RNAi element for a given receptor were crossed to elav-Gal4 females. Adult flies obtained from these crosses were brought to room temperature 3 days before the beginning of the following procedure. Total RNA from about 50 fly heads was retro-transcribed and cDNA obtained was subjected to PCR [33] using specific primers for transcripts of interest and the housekeeping gene GAPDH2. PCR protocol used is as follows: after 5 m at 94°C, samples were subjected to 35 cycles of 30 s at 94°C, then 30 s at 52°C, followed by 30 s at 72°C; after the last cycle, samples were incubated for 10 m at 72°C. Sequence of primers used was 5HT_1A_RF: 5′-GCCACTTCTGCCCATTTTGG-3′; 5HT_1A_RR: 5′-CCGATTGCCTTCTGGTGTCT-3′; 5HT_1B_RF: 5′-CAACGCCGAAGACTGAAAGC-3′; 5HT_1B_RR: 5′-CCGGGA-TGTGACAACGATGA-3′; 5HT_2_RF: 5′-TTACAGCCCTGAACACGACC-3′; 5HT_2_RR: 5′-CCAGTACGTCACACGTCACA-3′; 5HT_7_RF: 5′-AGTTTCTACGCGATTCGGCT-3′; 5HT_7_RR: 5′-GCGAATGCTGGTCGCAATTT-3′. The sequence of GAPDH2 primers was GAPDH2F: 5′-GCAAGGGTGCGTCCTATGAT-3′; GAPDH2R: 5′-AGAGTGTGGGTGGGTAGTGT-3′. In each case, it is first identified the forward and then the reverse primer.

The expression of a target gene is calculated for each sample as the expression ratio of the gene over the housekeeping gene [33]. The efficiency of RNAi procedure is calculated by measuring the change in this ratio in flies pan-neuronally expressing the RNAi as compared to flies that are not expressing the RNAi (undriven RNAi).

### Video Tracking

In the day of the experiment, a single third instar larva was collected, rinsed in tap water and placed on the middle of a 35 mm petri dish half-filled with 1% agar. The larva was allowed to freely move for 1 minute. Afterwards, larva movement was recorded for 140 secs (Olympus Digital Camera). To avoid the potential influence of external or visual cues on larval movement, the recordings were carried out under constant illumination in a closed box. Locomotion behavior was analyzed using an automated tracking system (Image-Pro Plus 6.0 software; Media Cybernetics Inc, Rockville, MD, USA), to measure distance covered by the animal (in mm).

### Drug Treatment

Flies were exposed to fluoxetine (100 µM, Tocris Bioscience, Ellisville, MO), alpha-methyl-L-Dopa (1 mM, Sigma-Aldrich Co, St Louis, MO) and MDMA (1 mM, a kind gift of Dr Patricio Saez-Briones, Universidad de Santiago, Chile) for 1 hr (as previously reported in [34]). Briefly, 200 µL of each drug were spread on the surface of an agar plate. Then, about 10–15 larvae were exposed to the drug for 1 hour. Next, a single third instar larva was collected, rinsed in distilled water and placed on an agar plate. Experiments were carried out as explained above. The concentrations of these drugs were chosen according to the information available in the literature [34]–[38] and preliminary experiments carried in our lab (data not shown). Control animals were treated in the same manner with solvent (water).

### Data Processing and Statistical Analysis

Values given are mean+SEM. Statistical comparisons (experimental versus genetic controls) were done using a one-way ANOVA followed by Tukey post hoc test. The significance level was set at P<0.05. All statistical studies were performed in GraphPad Prism 5.0 software (GraphPad Software, La Jolla, CA, USA).

### Biosafety Issues

The experimental procedures were approved by the Bioethical and Biosafety Committee of the Facultad de Ciencias Biológicas, Pontificia Universidad Católica de Chile and were conducted in accordance with the guidelines of the National Fund for Scientific and Technological Research (FONDECYT) and the Servicio Agrícola y Ganadero de Chile (SAG).

## Results

### 5HT System and Locomotion in *Drosophila* Larvae

In order to evaluate the contribution of 5HT systems to the generation of motor programs in fly larvae, we first assessed how different drugs that affect this system modify locomotion in *Drosophila* larvae.

Third-instar larvae are foraging animals and they are most of the time moving. Research groups use different systems and approaches to measure locomotion in fly larvae. For instance, in Saraswati et al (2004) [9] and Selcho et al (2012) [10], authors place a single larva on a gridded arena and evaluate the number of times the animal crosses one square. In Rodriguez Moncalvo & Campos (2009) [11], authors record the movement of larvae on a regular arena, digitalize their movement over 30 s and assess locomotion as the number of pixels traversed during the recording. We measure locomotion as the overall distance covered by larvae over the record time (140 sec), since this measurement provides information on the ability of animals to generate and execute motor programs. This is a method that has been previously reported [20], [39].

**Figure 1:**
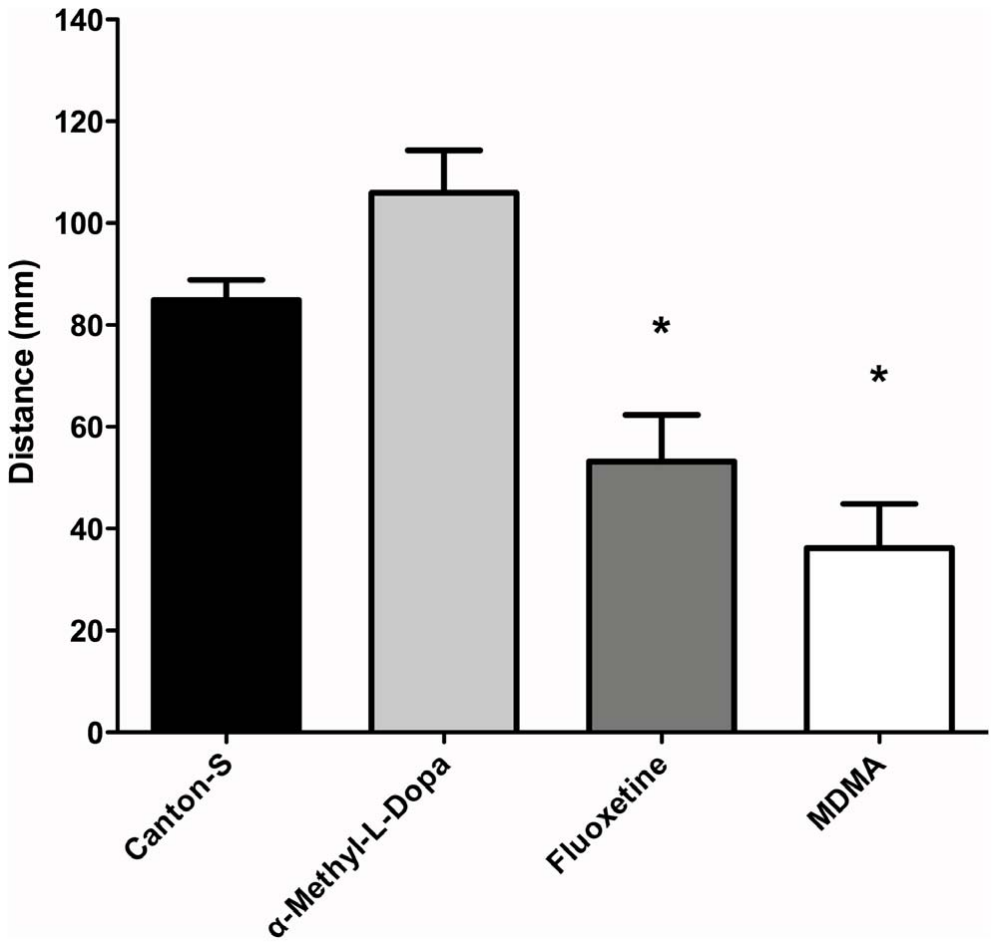
Pharmacological agents hindering 5HT neurotransmission affect locomotion in fly larvae. Canton-S flies were treated for 1 hr with α-methyl-L-Dopa (1 mM), Fluoxetine (100 µM) and MDMA (1 mM). After drug treatment, larval locomotion was evaluated. Data shown represent mean distance+SEM covered by n = 14 or more animals over 140 sec; * indicate p<0.05 compared to control Canton-S flies, ANOVA followed by Tukey post-hoc.

Acute treatment of animals with α-methyl-L-Dopa, a drug that inhibits the Dopa decarboxylase enzyme [36], induces an increased locomotion that does not reach statistical significance when compared to control animals (Fig. 1; 84.84±4.03 and 105.90±8.94 mm, in Canton-S animals in absence and presence of 1 mM α-methyl-L-Dopa, respectively. P>0.05, n = 14 animals). On the other hand, two drugs that acutely increase extracellular 5HT levels by blocking the serotonin transporter [37]–[38], [40], fluoxetine (100 µM) and MDMA (1 mM), decrease locomotion in *Drosophila* larvae compared to control Canton-S (Fig. 1). These data show that acute pharmacological manipulations of the 5HT system affect fly larval locomotion, suggesting an active role for 5HT in motor behavior.

**Figure 2:**
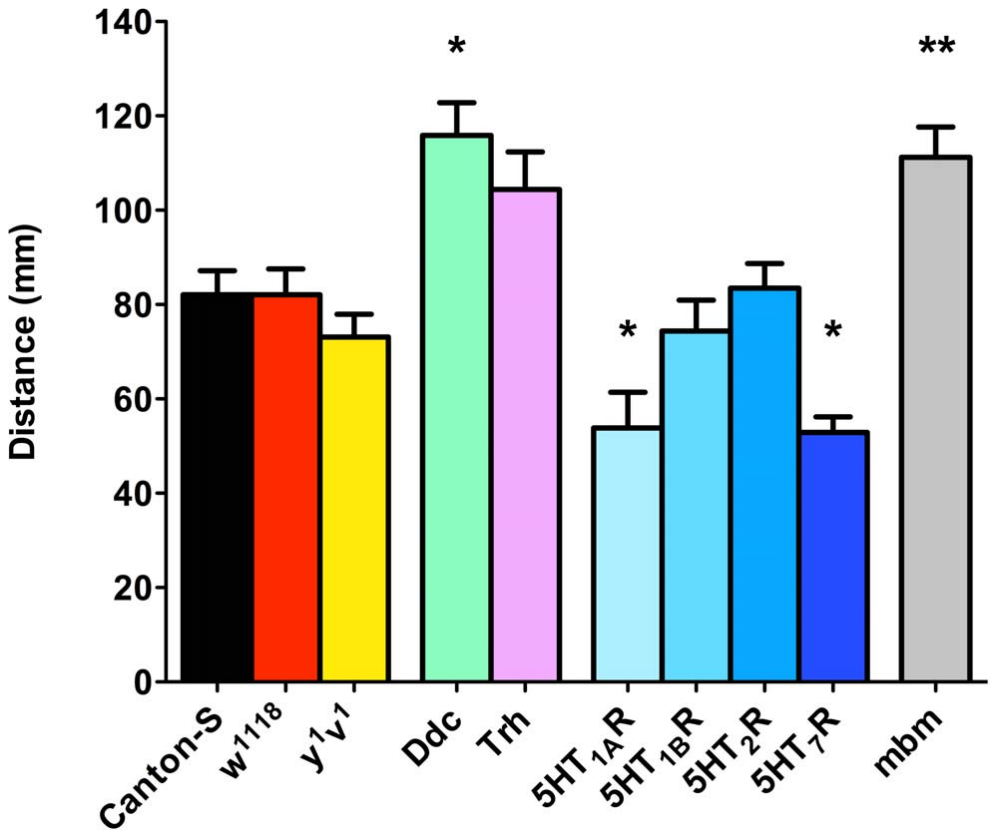
Animals expressing mutations in biosynthetic enzymes for 5HT and its receptors show impaired locomotion. Locomotion was studied in animals showing a single mutation in the biosynthetic enzymes Ddc and Trh, and the different 5HT receptors. Motor output was also evaluated in a mutant for MB development (mbm). Bars represent mean+SEM of at least 9 animals per experimental group. * indicate p<0.05 compared to Canton-S and *w^1118^* genetic control, and ** indicate p<0.01 compared to *y^1^v^1^* genetic control; ANOVA followed by Tukey post-hoc test.

We then decided to evaluate whether fly mutants in the 5HT biosynthetic pathways show impaired locomotion. Since the vast majority of animals expressing mutations for BA biosynthetic enzymes have been generated in a *w^1118^* background which is deficient in the *white* gene, a transporter for biomolecules such as guanine and tryptophan [41], or in a *y^1^v^1^* genetic background, we evaluated whether the genetic background affects motor behavior compared to one of the most commonly used wildtype strains, Canton-S. Results obtained show no differences in motor activity for *w^1118^* or *y^1^v^1^* larvae compared to Canton-S (Fig. 2).

Locomotion was then evaluated in animals expressing a mutation in Tryptophan hydroxylase (Trh), the 5HT rate-limiting biosynthetic enzyme, and Dopa decarboxylase (Ddc), the second enzyme in this biochemical pathway. Results show a roughly 30% increase in locomotion in larvae expressing a mutation in the Trh gene compared to the wildtype strain (82.09±5.48 and 104.40±7.99 mm in *w^1118^* and the Trh enzyme mutant, respectively), although it was not statistically significant (Fig. 2). On the other hand, larvae expressing a mutation in the Ddc enzyme show a significant increased locomotion (Fig. 2). These data show that manipulations that hinder 5HT biosynthesis increase motor output in the fly larva.

Next, we evaluated whether animals expressing mutations in any of the 5HT receptors cloned in *Drosophila* show alterations in their locomotor behavior. Results show that animals bearing mutations in 5HT_1A_R and 5HT_7_R genes show reduced locomotion compared to control flies, while no effect on locomotion is observed in mutants for 5HT_1B_R and 5HT_2_R genes (Fig. 2). In general, these results suggest that the serotonergic system modulate the execution of locomotor programs in *Drosophila* larva and suggest that 5HT receptors are differentially involved in this modulation. However, they do not clarify whether these effects are peripheral (e.g. muscle) or centrally mediated.

Our hypothesis is that 5HT receptors expressed in the brain, and particularly in the MB, could play a role in the modulation of motor programs. This hypothesis is supported by experiments carried out in a mutant for MB development (Fig. 2), where an increased locomotion is observed. We decided to further evaluate this issue, aiming at the possibility that these effects could depend upon specific 5HT receptors in MB.

To do this, we employed the Gal4-UAS technique to pan-neuronally express RNAi directed to the transcripts of each of the cloned 5HT receptors in *Drosophila* larvae by using the elav-Gal4 driver. We also utilized Gal4 drivers directing the expression of genes to the whole population of neurons in MB (OK107-Gal4) or to MB neuronal subpopulations: 201y-Gal4 for MB γ lobe-forming neurons and c305a-Gal4 for the larval-born MB α’/β’ neurons [42]. The efficiency of the RNAi tools in decreasing the expression of the transcripts for the different genes was 48.78±4.09, 58.74±15.93, 65.03±12.85 and 66.88±12.39 for the 5HT_1A_R, 5HT_1B_R, 5HT_2_R and 5HT_7_R, respectively. We believe this is an underestimation of the potency of the genetic tools we used: the RNAis were expressed only in CNS neurons while the evaluation of the efficacy of the RNAi transgenes was carried out in fly heads, a biological material that contains other cell types.

### 5HT Receptors Expressed in MB Differentially Contribute to Motor Programs in *Drosophila* Larvae

**Figure 3:**
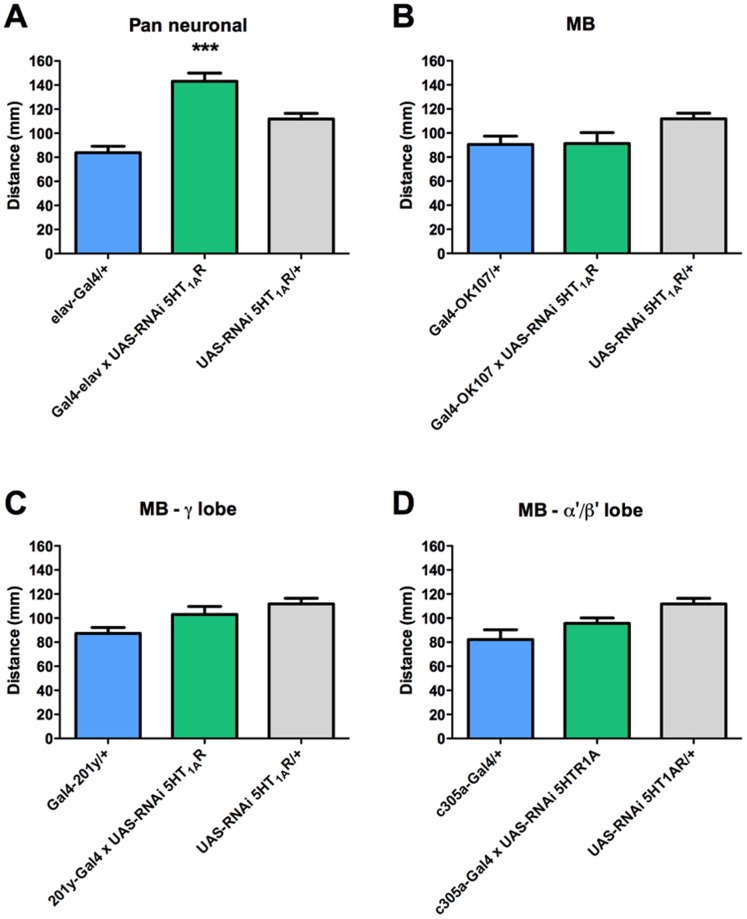
CNS effect of 5HT_1A_R on locomotion does not depend on MB. A. Pan neuronal expression of RNAi for 5HT_1A_R (Gal4-elav x UAS-RNAi 5HT_1A_R, in green) increases larval locomotion compared to genetic controls (elav-Gal4/+, in blue, and UAS-RNAi 5HT_1A_R/+, in gray). Expression of RNAi for 5HT_1A_R in the entire MB neuronal population (B.), or in the γ (C.) or α’/β’ (D.) lobe-forming neurons (all in green) do not affect larval locomotion compared to Gal4 (in blue) and UAS-RNAi 5HT_1A_R/+ (in gray) genetic controls. Bars represent mean distance+SEM that at least 9 larvae covered over 140 sec, per experimental condition. *** indicates p<0.001 compared to genetic controls; ANOVA followed by Tukey post-hoc test.

Expression of RNAi directed to 5HT_1A_R mRNA under the control of the pan neuronal elav-Gal4 driver induces a dramatic increase in locomotion as compared to genetic controls (Fig. 3A). Interestingly this increased motor activity is not observed when driving the expression of the RNAi for 5HT_1A_R with any of the MB-Gal4 lines (Fig. 3B–D). This data suggest that the neural substrate for the effect of 5HT_1A_R on locomotion is a brain region different from the MB.

**Figure 4:**
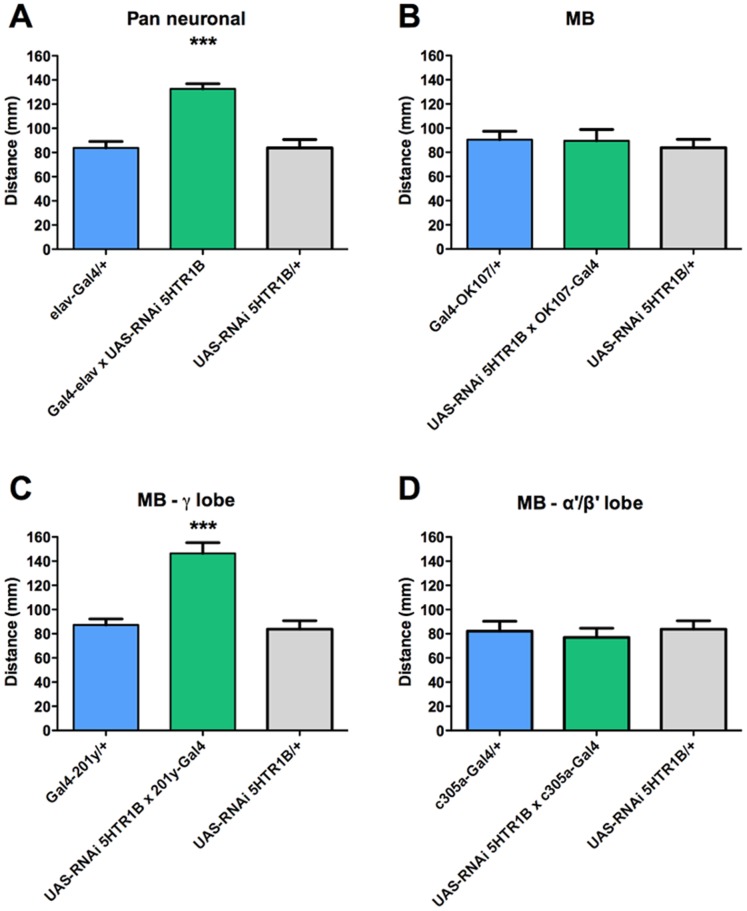
Increased locomotion observed in animals pan-neuronally expressing RNAi for 5HT_1B_R depends on MB γ-lobe forming neurons. A. Pan neuronal expression of RNAi for 5HT_1A_R (Gal4-elav x UAS-RNAi 5HT_1B_R, in green) increases larval locomotion compared to genetic controls (elav-Gal4/+, in blue, and UAS-RNAi 5HT_1A_R/+, in gray). B. Expression of RNAi for 5HT_1A_R in the entire MB neuronal population (in green) does not affect motor output, as compared to genetic controls. C. Expression of RNAi for 5HT_1A_R in the γ-lobe forming neurons increases locomotion (in green) compared to genetic controls. D. No effect on locomotion is observed when the RNAi for 5HT_1A_R ισ εξπρεσσεδ ιν τηε α’/β’ lobe-forming neurons compared to its genetic controls. In each case, genetic controls are animals bearing one copy of the Gal4 driver (in blue) or the undriven UAS-RNAi 5HT_1A_R (in gray). Bars represent mean distance+SEM that at least 9 larvae covered over 140 sec, per experimental condition. *** indicates p<0.001 compared to genetic controls; ANOVA followed by Tukey post-hoc test.

Pan neuronal expression of the RNAi directed to the transcript for the other 5HT type 1 receptor, 5HT_1B_R, increases locomotion in fly larvae (approximately 50%) compared to the genetic controls (Fig. 4A). Interestingly, although the expression of the RNAi for 5HT_1B_R under the control of the general MB driver fails to show any effect on locomotion (Fig. 4B), when this RNAi is expressed under the control of the γ-lobe specific 201y-Gal4 driver, a significant increase in locomotion is detected compared to its genetic controls (Fig. 4C), suggesting that the 5HT_1B_R expressed in this neuronal MB subpopulation could be at least partially involved in the generation of this motor behavior. On the other hand, driving the expression of the RNAi for 5HT_1B_R under the control of the α’/β’-lobe specific c305a-Gal4 driver show no effect on locomotion (Fig. 4D).

**Figure 5:**
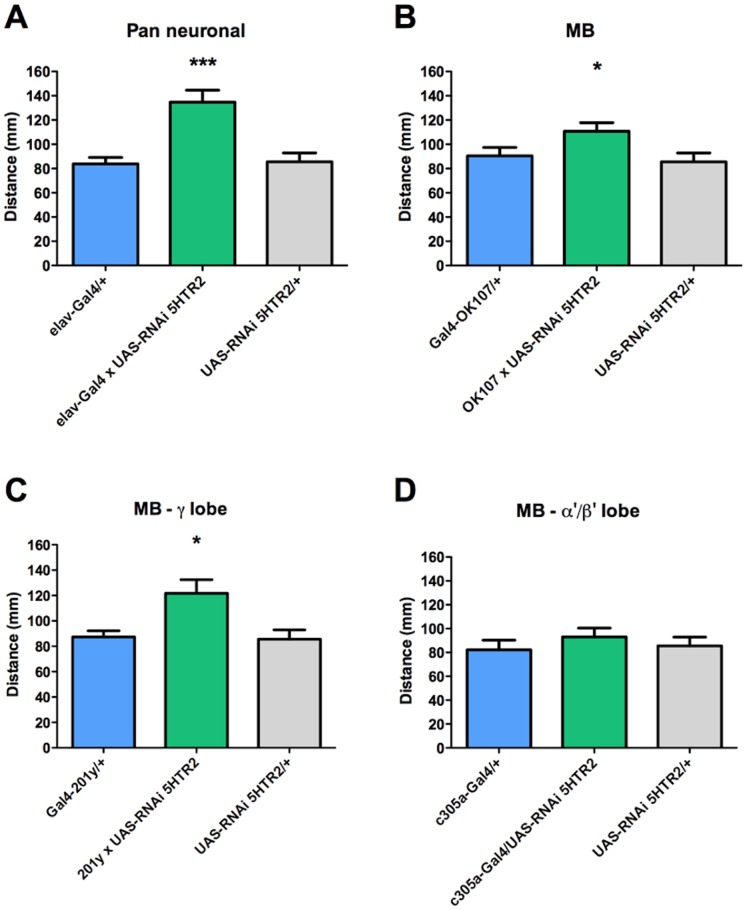
Increased locomotion in animals expressing RNAi for 5HT_2_R in the entire MB neuronal population or in the γ-lobe forming neurons. The expression of RNAi for 5HT_2_R pan-neuronally (A.), in the entire MB (B.) or in the γ-lobe forming neurons (C.) increase locomotion (all in green), compared to genetic controls (in blue and gray). No effect on locomotion is observed when the RNAi for 5HT_2_R is expressed in the α’/β’ MB forming neurons compared to genetic controls (D.). In each case, genetic controls are animals bearing one copy of the Gal4 driver (in blue) or the undriven UAS-RNAi 5HT_2_R (in gray). Bars represent mean distance+SEM that at least 9 larvae covered over 140 sec, per experimental condition. * and *** indicates p<0.05 and p<0.001 compared to genetic controls; ANOVA followed by Tukey post-hoc test.

Expression of the RNAi directed against 5HT_2_R mRNA in the CNS induces an increase in locomotor activity (Fig. 5A), which is also observed when this RNAi is expressed under the control of OK107 (Fig. 5B) and 201y (Fig. 5C) gal4 drivers. No effect in locomotion is observed after the expression of the RNAi for 5HT_2_R under the control of the c305a-Gal4 driver (Fig. 5D). These data suggest that the effect of the pan-neuronal expression of the RNAi for 5HT_2_R on locomotion is at least partly explained by effects on MB neurons.

**Figure 6:**
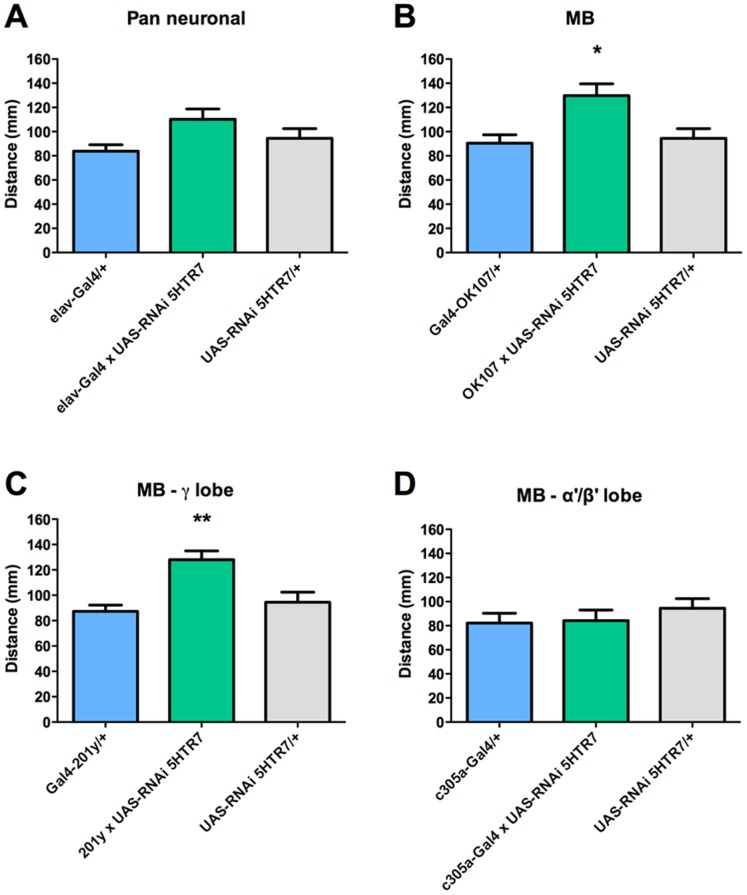
5HT_7_R expressed in the MB modulate locomotion. A. Pan-neuronal expression of RNAi for 5HT_7_R does not affect locomotion (in green) compared to genetic controls. Expression of RNAi for 5HT_7_R in the entire MB neuronal population (B.) or in the MB γ-lobe forming neurons (C.) increases locomotion, while no effect is observed when the RNAi for this receptor is expressed in the α’/β’ lobe forming neuronal subpopulation (D.). In each case, genetic controls are animals bearing one copy of the Gal4 driver (in blue) or the undriven UAS-RNAi 5HT_7_R (in gray). Bars represent mean distance covered by animals over 140 sec+SEM, n = 9 animals or more. * and ** indicates p<0.05 and p<0.005 compared to genetic controls; ANOVA followed by Tukey post-hoc test.

Finally, the pan neuronal expression of RNAi for 5HT_7_R does not show any effect on locomotion (Fig. 6A). However, the expression of this RNAi in the entire MB population by using the OK107-Gal4 driver (Fig. 6B) or in the MB γ-lobe neuronal subpopulation with the use of 201y-Gal4 driver (Fig. 6C), results in an increased locomotion behavior as compared to the respective genetic controls. No effect on locomotion is observed when the RNAi for the 5HT_7_R is expressed under the control of c305a-Gal4 driver (Fig. 6D). These data suggests that 5HT_7_R-expressing MB neurons are also involved in larval locomotion.

Overall these results suggest that 5HT receptors expressed in MB γ-lobes play an important role in the modulation of motor output. These data are further supported by experiments carried out using a different driver line, MB247-Gal4 (Fig. S1), which in fly larvae only labels the γ-lobe [42].

## Discussion

### Locomotion and 5HT Systems

Animals need to tightly control their movements so they adequately respond to the vast variety of stimuli they are exposed to in their environment. It has been shown that the thoracic ganglia of insects have an important role in establishing a basic motor plan that can be centrally orchestrated by specific regions of the invertebrate brain, particularly the MB and CC [12], [13], [43]–[47]. Interestingly, BA systems play an important role in the execution of insect motor programs by acting on the motor neurons and the muscles they innervate [48]–[50]. They also contribute in the planning and execution of these motor programs through their actions in the insect brain, as it has been shown for the 5HT and DA systems ([11] and [2], respectively), possibly by modifying the activity of neurons in the CC and MB. Consistent with this idea, it has been shown that the motor responses associated to two different stimuli depend on the dopaminergic system and the type 1 DA receptor expressed in the adult fly CC [51]–[52].

Neural BA systems in the fly are complex: aminergic systems innervate different brain regions; each neuroactive molecule has a specific set of receptors that mediate their actions; and their synthesis and release is controlled by precise and unique mechanisms [22], [53]–[55]. Unfortunately there is little information on how the different BA receptors expressed in brain regions of interest modulate motor programs in insects. Here we decided to begin evaluating this issue by assessing the contribution of the 5HT neural system and its receptors expressed in MB to *Drosophila* larval locomotion.

We first assessed whether pharmacological agents that interfere with the 5HT system, α-methyl-L-Dopa, fluoxetine and MDMA, were able to modulate locomotion in fly larvae. Results obtained show that two drugs that increase 5HT extracellular levels, fluoxetine and MDMA, reduce locomotion in flies. On the other hand, larvae in presence of α-methyl-L-Dopa, a drug that hinders 5HT synthesis, show increased locomotion. Therefore, these data suggest that 5HT systems inhibit motor programs in fly larvae.

To further confirm our pharmacological data, we decided to evaluate whether flies expressing mutations in the enzymes responsible for 5HT synthesis were affected in locomotion. Results obtained show that mutants in Trh, the key protein in 5HT biosynthesis, show an increased locomotion that does not reach statistical significance when compared to control strains, possibly due to the high level of variability between measurements. However, locomotion is statistically increased in mutants for the second enzyme in 5HT biosynthesis, Ddc. This enzyme also contributes to the biosynthesis of DA, but as it has been previously suggested, this amine would have no contribution to larval locomotion [11], [34]. Thus, the most likely explanation is that the increased locomotion observed in these mutants is explained by the modification of 5HT levels.

Overall, the data obtained with these pharmacological and genetic tools show that an increase in 5HT levels induces a decrease in locomotion, while a decrease in the amine levels enhances motor output. These data are in agreement with our suggestion that 5HT is an important regulator of locomotion in flies, inhibiting motor programs. This is also in agreement with previous data obtained in other invertebrates (e.g. the worm *Caenorhabditis elegans;* [56]) and vertebrates [57] demonstrating that the contribution of this amine to motor control is highly conserved throughout evolution.

### 5HT Receptors Differentially Modulate Locomotion

As an alternative approach to evaluate the contribution of 5HT systems on locomotion in fly larva, we decided to assess whether animals expressing mutations in any of the 5HT receptors described in *Drosophila* show motor deficiencies. Our results show that mutants for 5HT_1A_R and 5HT_7_R exhibit reduced locomotion compared to their controls. The animals expressing mutations for the other two receptors (5HT_1B_R and 5HT_2_R) do not show any alteration in motor behavior. These results further argue in favor that 5HT systems are important modulators of motor programs in flies. However, do not say much about the anatomical site where 5HT receptors are exerting their action. This is an important issue, considering that several studies have shown that neural systems present in peripheral tissues and in the CNS could differentially modulate locomotion [11].

In order to directly evaluate the contribution of 5HT receptors expressed in the fly CNS to motor behavior in larvae, we used several Gal4 driver lines to pan-neurally express RNAi directed to the different 5HT receptors. It is noteworthy that most of the results obtained when evaluating locomotion in animals expressing mutations for 5HT receptors, are different to those obtained when expressing the RNAi for the different receptors in the whole CNS. For instance, our results with the RNAi for 5HT_1A_R show increased locomotion while the results obtained in the animals expressing a mutation for this receptor showed the opposite effect. These data would argue in favor that 5HT_1A_ receptors expressed in peripheral tissues and in the CNS have opposite effects on motor programs in the fly larvae. A similar phenomenon is possible to be postulated for 5HT_1B_R and 5HT_2_R: pan neuronal expression of an RNAi for these receptors increase locomotion compared to controls, which suggests that these receptors inhibit motor programs in fly larvae. However, animals expressing mutations for these receptors show no alteration in locomotion, which suggests that CNS and peripheral 5HT_1B_R and 5HT_2_R receptors would be exerting opposite effects on motor programs in the fly larvae. On the other hand, while the mutant for 5HT_7_R show overall decreased locomotion, the pan neuronal expression of an RNAi for 5HT_7_R showed no effect on locomotion. Overall, these results support the proposition that peripheral and CNS 5HT systems differentially modulate motor programs [11].

### 5HT Receptors Expressed in MB Differentially Modulate Locomotion

Since our data suggest that 5HT receptors expressed in the larval brain would be responsible for the modulation of locomotion in the animal, we decided to evaluate whether the site of such regulation is the MB. This hypothesis is supported by our data showing that a mutant for MB development show increased motor output, and is consistent with previous reports in the literature [21].

Results obtained when expressing an RNAi for 5HT_1A_R in the whole MB region show no effect on locomotion compared to genetic controls. This was further corroborated using Gal4 lines for specific MB subpopulations. Thus, these data suggest that the increase in locomotion observed in animals expressing the RNAi for 5HT_1A_R in the entire CNS would be explained by the effect of this receptor on a different fly brain region, possibly CC. This is different from what we observe for the other 5HT receptor types, which demonstrates the heterogeneity of the effects of the various 5HT receptors on larval locomotion.

The neurons forming the MB are diverse in their origin and anatomical organization. Adult fly MB neurons are classified according to their axonal projections into different lobes: α/β, α’/β’ and γ type neurons. Interestingly, it has been suggested that this anatomical organization has functional consequences. For instance, the adult γ-lobe is associated to short-term memory, while the neurons forming the adult α/β ανδ α'/β’-lobes are associated to long-term memory [58]. Larval MB is less complex. Actually, the MB at this developmental stage is mostly accounted for neurons of embryonic origin that persist to the adult stage to become part of the γ-lobes [30], [41]. In contrast, the neurons that at the adult stage form the α’/β’-lobe are born when the animal is at the larval stages. Therefore, it is possible to ask whether these two larval MB subpopulations, α’/β’ and γ-lobe, are playing a role in the effects observed in our locomotion assays. In order to evaluate this question, we used three different Gal4 lines, OK107-Gal4, c305a-Gal4 and 201y-Gal4 that target the entire larval MB population, the α’/β’ and the γ lobes, respectively [41]. Driving the RNAi for the different 5HT receptors in the α’/β’ lobe subpopulation did not induce any change in larval locomotion, which would be consistent with the idea that this neuronal population does not play a role in the generation or execution of motor programs at this developmental stage. Thus, the effects on locomotion associated to the expression of RNAi for the different 5HT receptors in the entire MB population could be attributed to the neurons that will constitute the γ-lobe neuronal subpopulation in the adult brain. This proposition is consistent with the data we obtained when expressing the RNAi for 5HT_1B_R, 5HT_2_R and 5HT_7_R in the entire MB population or in the γ-lobe only.

**Figure 7:**
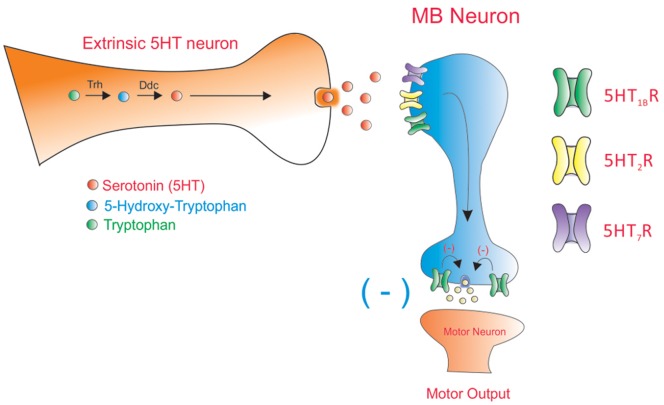
A model to explain the contribution of 5HT receptors expressed in MB to larval motor programs. 5HT_2_R and 5HT_7_R expressed in MB would receive serotonergic information arriving to MB from extrinsic neurons. When activated by these receptors MB would be able to exert its inhibitory role on motor programs. On the other hand, 5HT_1B_R would be expressed in presynaptic and postsynaptic domains, where they could exert different actions on MB neuronal excitability and/or in the control of the communication between MB and downstream neurons. Thus, the sum of effects of 5HT_1B_R at different levels in MB would account for the effect of this receptor on motor output.

Although very few studies exist on the cellular responses generated by activation of 5HT receptors in fly neurons, sequence homology and functional studies in heterologous systems suggest that *Drosophila* 5HT_1B_R exerts inhibitory effects on intracellular signaling pathways. Expression studies suggest this 5HT receptor subtype could be found both at presynaptic and postsynaptic domains [55], [59]. Thus, it is possible to suggest that the contribution of 5HT_1B_R to motor programs would be explained by the sum of effects induced by this receptor at different levels on MB (Fig. 7).

It is a puzzling observation that no effects on locomotion are observed when the OK107-Gal4 element drives the expression of the RNAi directed to 5HT_1B_R, while a clear and robust effect is observed when using the 201y-Gal4 and MB247-Gal4 driver lines. Our data on the efficiency of the different RNAis in decreasing the expression of their target genes support the idea that all the RNAi tools we used are effective at doing so, and therefore we do not believe this is the explanation for this finding. Although it has been shown that OK107-Gal4 is a strong driver for MB, it is also efficient at driving the expression of genes in other brain regions in the adult fly brain [30]. As pointed out by Pauls et al [42], there are not too many studies comparing the expression of Gal4 drivers in flies at the larval and the adult stage, but it is highly likely that the OK107-Gal4 is driving the expression of the RNAi for 5HT_1B_R in a larval brain region different from the MB. Thus, it is possible to speculate that the opposite contribution of 5HT_1B_R in MB and other brain region(s) determine that no effect on the final motor output is detected when using the OK107-Gal4 driver. Interestingly, this is only observed in experiments evaluating the contribution of 5HT_1B_R, which further suggests that only this receptor would be expressed and/or is relevant in the other brain region(s) for motor control.

On the other hand, it has been suggested that 5HT_2_R and 5HT_7_R are coupled to intracellular excitatory signaling cascades as their vertebrate counterparts [55]. Although no exhaustive information is available on 5HT receptor expression at larval stages, data in the literature suggest that 5HT_2_R and 5HT_7_R are postsynaptic receptors expressed at some extent in MB [55], [60]–[61]. Thus, it is possible to suggest that these receptors are postsynaptic to the serotonergic neurons that arrive and innervate MB [22], [62]. Our data support a model in which 5HT_2_R and 5HT_7_R would activate MB neurons to exert the inhibitory control on motor programs associated to this brain region (Fig. 7).

In conclusion, our data suggest that CNS 5HT systems are important regulators of motor behavior in Drosophila *larvae* and that 5HT receptors expressed in fly MB differentially participate in the control of motor output in these animals.

## Supporting Information

Figure S1 **Expression of RNAi for 5HT_1B_, 5HT_2_ and 5HT_7_ receptors in MB γ-lobe neurons using a different Gal4 driver line cause an increased in motor output.** The different RNAi were expressed under the control of MB247-Gal4, a driver line that only labels γ-lobe neurons in flies at the larval stage (Pauls et al, 2010). Results show that only 5HT_1B_, 5HT_2_ and 5HT_7_ receptors increase locomotion, while no effect is observed in animals expressing the RNAi for 5HT_1A_ in MB γ-lobe. These data further confirm our hypothesis that this MB neuronal subpopulation is responsible for the effects of this manipulation on locomotion. Data shown represents mean+SEM of at least 9 different animals. *, **, ***, indicate p<0.05 compared to respective controls.
(TIFF)
