# Supplementary figures and images for: Serotonin Receptors Expressed in Drosophila Mushroom Bodies Differentially Modulate Larval Locomotion
Source: PLoS One. 2014 Feb 25;9(2):e89641. doi: 10.1371/journal.pone.0089641 (PMC3934909; doi:10.1371/journal.pone.0089641)

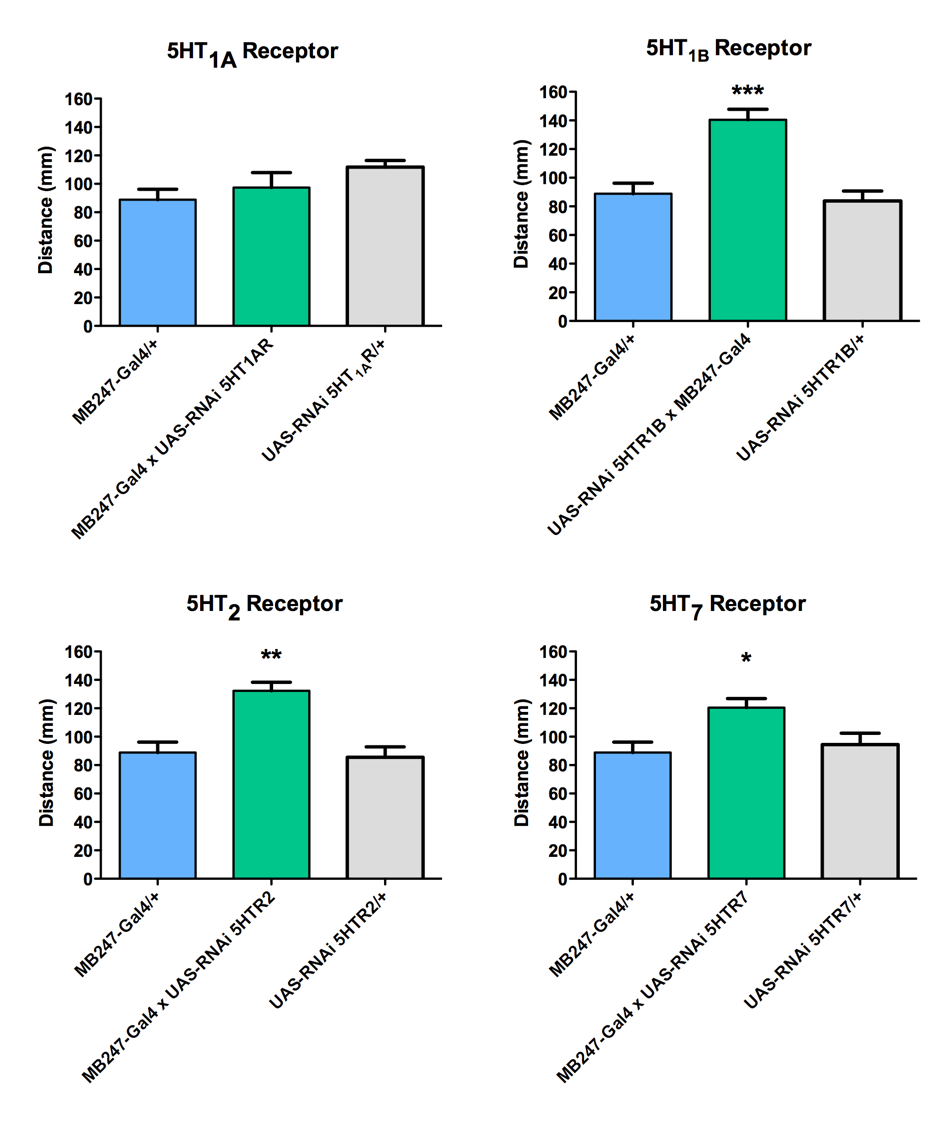

Supplement: Figure S1 — Expression of RNAi for 5HT1B, 5HT2 and 5HT7 receptors in MB γ-lobe neurons using a different Gal4 driver line cause an increased in motor output. The different RNAi were expressed under the control of MB247-Gal4, a driver line that only labels γ-lobe neurons in flies at the larval stage (Pauls et al, 2010). Results show that only 5HT1B, 5HT2 and 5HT7 receptors increase locomotion, while no effect is observed in animals expressing the RNAi for 5HT1A in MB γ-lobe. These data further confirm our hypothesis that this MB neuronal subpopulation is responsible for the effects of this manipulation on locomotion. Data shown represents mean+SEM of at least 9 different animals. *, **, ***, indicate p<0.05 compared to respective controls. (TIFF) [file pone.0089641.s001.tiff]
